# Comprehensive analysis for genetic diagnosis of Dystrophinopathies in Japan

**DOI:** 10.1186/s13023-017-0703-4

**Published:** 2017-08-31

**Authors:** Mariko Okubo, Kanako Goto, Hirofumi Komaki, Harumasa Nakamura, Madoka Mori-Yoshimura, Yukiko K. Hayashi, Satomi Mitsuhashi, Satoru Noguchi, En Kimura, Ichizo Nishino

**Affiliations:** 1 Department of Neuromuscular Research, National Institute of Neuroscience, National Center of Neurology and Psychiatry (NCNP), 4-1-1 Ogawa-Higashi, Kodaira, Tokyo, 187-8502 Japan; 2 Department of Pediatrics, Graduate School of Medicine and Faculty of Medicine, The University of Tokyo, Tokyo, Japan; 3 Department of Genome Medicine Development, Medical Genome Center, NCNP, Tokyo, Japan; 4 Department of Child Neurology, National Center Hospital, NCNP, Tokyo, Japan; 5 Department of Neurology, National Center Hospital, NCNP, Tokyo, Japan; 6 Department of Pathophysiology, Tokyo Medical University, Tokyo, Japan; 7 Department of Promoting Clinical Trial and Translational Medicine, Translational Medical Center, NCNP, Tokyo, Japan

**Keywords:** Duchenne and Becker muscular dystrophies, Genetic diagnosis, MLPA, Nucleotide sequencing

## Abstract

**Background:**

Duchenne muscular dystrophy (DMD) is the most common disease in children caused by mutations in the *DMD* gene, and DMD and Becker muscular dystrophy (BMD) are collectively called dystrophinopathies. Dystrophinopathies show a complex mutation spectrum. The importance of mutation databases, with clinical phenotypes and protein studies of patients, is increasingly recognized as a reference for genetic diagnosis and for the development of gene therapy.

**Methods:**

We used the data from the Japanese Registry of Muscular Dystrophy (Remudy) compiled during from July 2009 to March 2017, and reviewed 1497 patients with dystrophinopathies.

**Results:**

The spectrum of identified mutations contained exon deletions (61%), exon duplications (13%), nonsense mutations (13%), small deletions (5%), small insertions (3%), splice-site mutations (4%), and missense mutations (1%). Exon deletions were found most frequently in the central hot spot region between exons 45–52 (42%), and most duplications were detected in the proximal hot spot region between exons 3–25 (47%). In the 371 patients harboring a small mutation, 194 mutations were reported and 187 mutations were unreported.

**Conclusions:**

We report the largest dystrophinopathies mutation dataset in Japan from a national patient registry, “Remudy”. This dataset provides a useful reference to support the genetic diagnosis and treatment of dystrophinopathy.

**Electronic supplementary material:**

The online version of this article (10.1186/s13023-017-0703-4) contains supplementary material, which is available to authorized users.

## Background

Dystrophinopathies are an X-linked disease caused by mutations in the DMD gene (OMIM 300377) located at Xp21.2. *DMD* is a large gene, spanning more than 2.2 Mb of genomic DNA, and containing 79 exons and lengthy introns, which produces a 14-kb mRNA transcript [1]. Dystrophinopathies are the most common muscle disease in children which affects one in 3600–6000 live male births [2, 3], and has been classically classified into two forms: Duchenne muscular dystrophy (DMD; OMIM 310200) and Becker muscular dystrophy (BMD; OMIM 300376). DMD shows a severe phenotype clinically characterized by rapid progression in early childhood, and loss of ambulation during the second decade of life, while BMD shows a milder form with patients being ambulant after 16 years of age. Patients with an intermediate phenotype are sometimes referred to as having intermediate muscular dystrophy (IMD). Nevertheless, the classification into the three forms is not always easy.

To date, approximately 70% of mutations found in DMD patients are deletions/duplications of one or more exons, while the remaining 30% are caused by small mutations at the nucleotide level. Multiplex ligation-dependent probe amplification (MLPA), which can examine the duplications and/or deletions of all 79 exons, has been developed and widely used. [4–6] However, the remaining 30% of patients harboring small mutations are undiagnosed and need to perform sanger sequencing for diagnosis.

Recently, promising mutation-specific molecular therapies have been developed. For instance, exon skipping is expected to be applicable to patients that have one or more exons deletions in *DMD*. Furthermore, read-through therapy of a nonsense codon to produce full-length dystrophin is applicable to patients with DMD harboring nonsense mutations. Nonsense mutations are present in approximately 15% of all DMD patients [7–9]. However, the precise analysis of the *DMD* mutation is required for the application of these molecular therapies to patients.

In Japan, a hospital based dataset of 127 patients harboring small mutations by Takeshima et al. in 2010 [10], and a registry based dataset in Japanese Registry of Muscular Dystrophy (Remudy) of exon deletions and duplications by Nakamura et al. [11] have been compiled. Here, we extend these previous reports not only by increasing the number of the patients (1497 patients), but also by comprehensive genetic analysis of exonic and small nucleotide mutations and immunostaining of dystrophin on muscle biopsies to deduce genotype-phenotype correlations in patients with dystrophinopathies.

## Methods

### Registry-based datasets

Remudy was developed in 2009 in collaboration with the Translational Research in Europe-Assessment and Treatment of Neuromuscular Diseases (TREAT-NMD) Network of Excellence [11–13]. The Remudy database for male patients with dystrophinopathies includes clinical and molecular genetic data, as well as all mandatory and highly encouraged items for the TREAT-NMD global patient registry. To classify into DMD, BMD or IMD, the attending physicians reviewed their clinical information. Then, our clinical and genetic curators independently evaluated physicians’ classification by reviewing the clinical information, and pathological data (including dystrophin immune-staining, if applicable), and report of genetic analysis for *DMD*. In the present study, we used the registry data compiled during from July 2009 to March 2017.

### Analysis of small mutations

When the mutations were not detected by MLPA, but dystrophin immunostaining supported the diagnosis of dystrophinopathies, the nucleotide sequence of all exons and their flanking intronic regions in *DMD* was determined by IonPGM (Thermo Fisher Scientific, MA, USA) [14] or Sanger method. Missense variants were filtered with an allele frequency under 0.01 in the Human Genetics Variation Browser (HGVD; http://www.hgvd.genome.med.kyoto-u.ac.jp/), the Exome Aggregation Consortium (ExAC; http://exac.broadinstitute.org/), and the NHLBI Exome Sequencing Project (ESP6500; http://evs.gs.washington.edu/EVS/). In silico analysis of the effect of the amino acid change was conducted using Polymorphism Phenotyping version 2 (PolyPhen 2; http://genetics.bwh.harvard.edu/pph2/) [15, 16].

### Dystrophin immunostaining

Immunohistochemical analyses for dystrophin on patient’s muscle sections were performed using mouse monoclonal antibodies against dystrophin C-terminus (NCL-DYS2), rod (NCL-DYS1), and N-terminus (NCL-DYS3) (all from Novocastra Lab) by standard procedures as reported previously [17].

## Results

In July 2009 through March 2017, a total of 1497 dystrophinopathies patients were registered in Remudy. Among them, 1167, 295, and 35 patients were respectively diagnosed having DMD, BMD, and IMD. The genetic diagnosis was made by MLPA and/or multiplex-PCR in 1092 patients with exon deletion in 901 patients (61% of 1497 dystrophinopathies patients), exon duplications in 188 (13%), and both exon deletions and duplications in 3 (0.2%). All patients harboring single exon deletion were performed sanger sequencing to confirm the deletion.

Among the remaining 405 patients, DNA sequencing revealed small mutations in 371.

One patient was found to have pericentric inversion of the X chromosome by chromosome testing. One patients were diagnosed as having facioscalpulohumeral muscular dystrophy after registration. However, the remaining 32 patients remained genetically undiagnosed.

### Large deletion/duplication mutation patterns and hot spot analysis

**Fig. 1:**
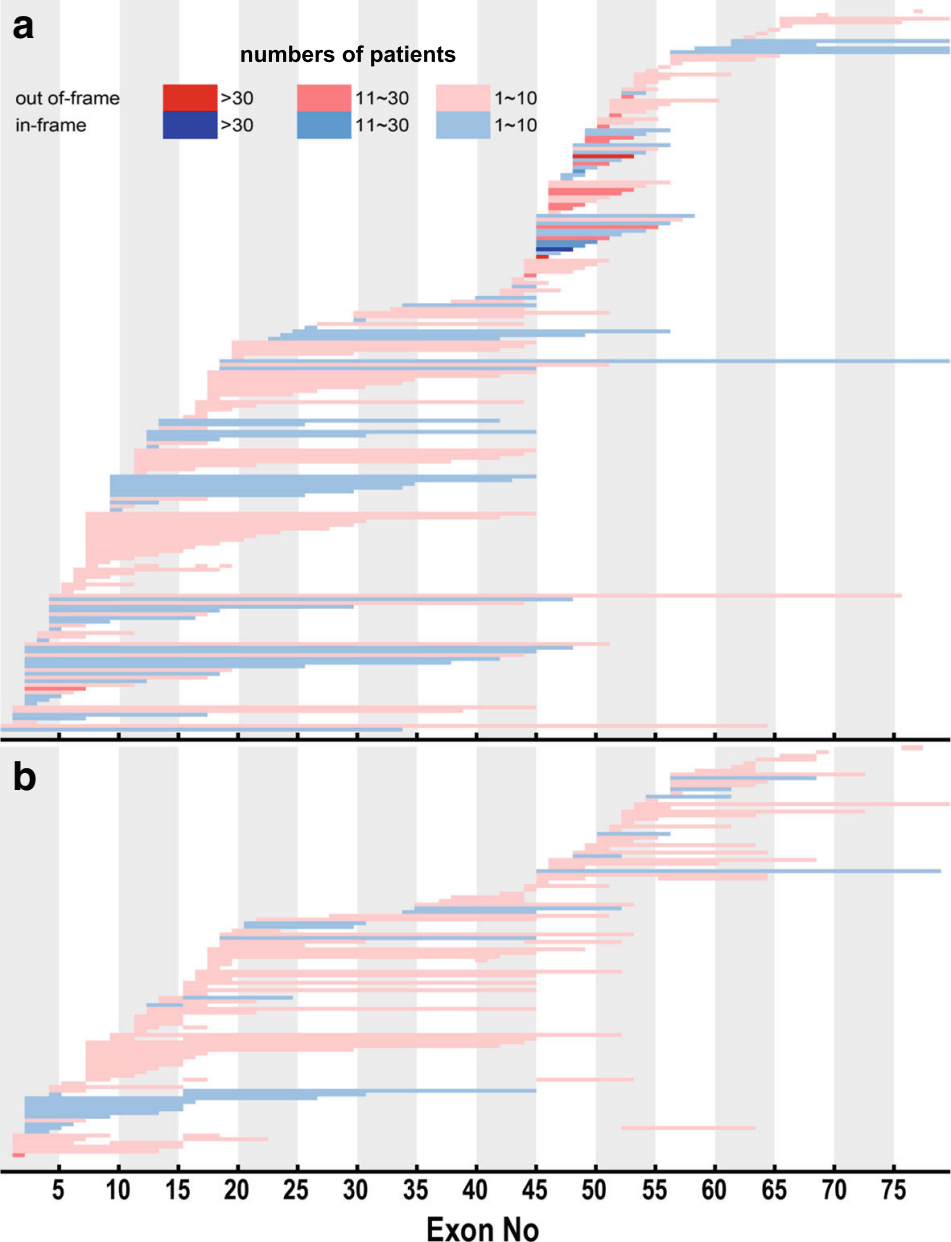
Patterns of exon deletion and exon duplication in the dystrophin gene in patients with dystrophinopathy. **a** Exon deletion. Each bar represents a deleted exon observed in a patient. **b** Exon duplication. Each bar represents a duplicated exon observed in a patient

**Fig. 2:**
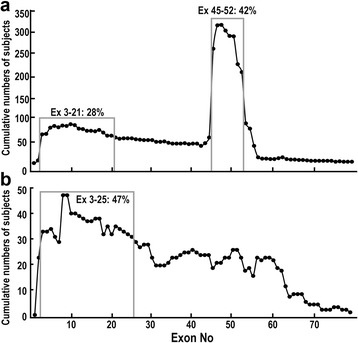
Cumulative numbers of subjects with a deletion/duplication per exon. **a** Deletions. A hot spot region is visible between exon 45 and exon 52. **b** Duplications. A hot spot region is visible between exon 3 and exon 25

The deletion or duplication patterns and their frequencies were plotted, in Fig. 1a and b. The cumulative number of subjects with deletions or duplications of each exon was plotted as shown in Fig. 2a and b. The most common exon deletion pattern was the deletion of exons 45–47 (68/901, 7.5%) (Fig. 1a). Most exon deletions in patients with dystrophinopathies were observed in the hotspot region between exons 45 and 52 (42%) (Fig. 2a). A second frequent-deletion hotspot region towards the 5′-end was distinguished in the region between exons 3 and 21 (28%) (Fig. 2a). On the other hand, the most common exon duplication pattern was the duplication of exon 2 (14/188, 7.4%) (Fig. 1b). The largest duplications in the patients with dystrophinopathies were observed in the hotspot region between exons 3 and 25 (47%) (Fig. 2b).

### Small mutations

**Fig. 3:**
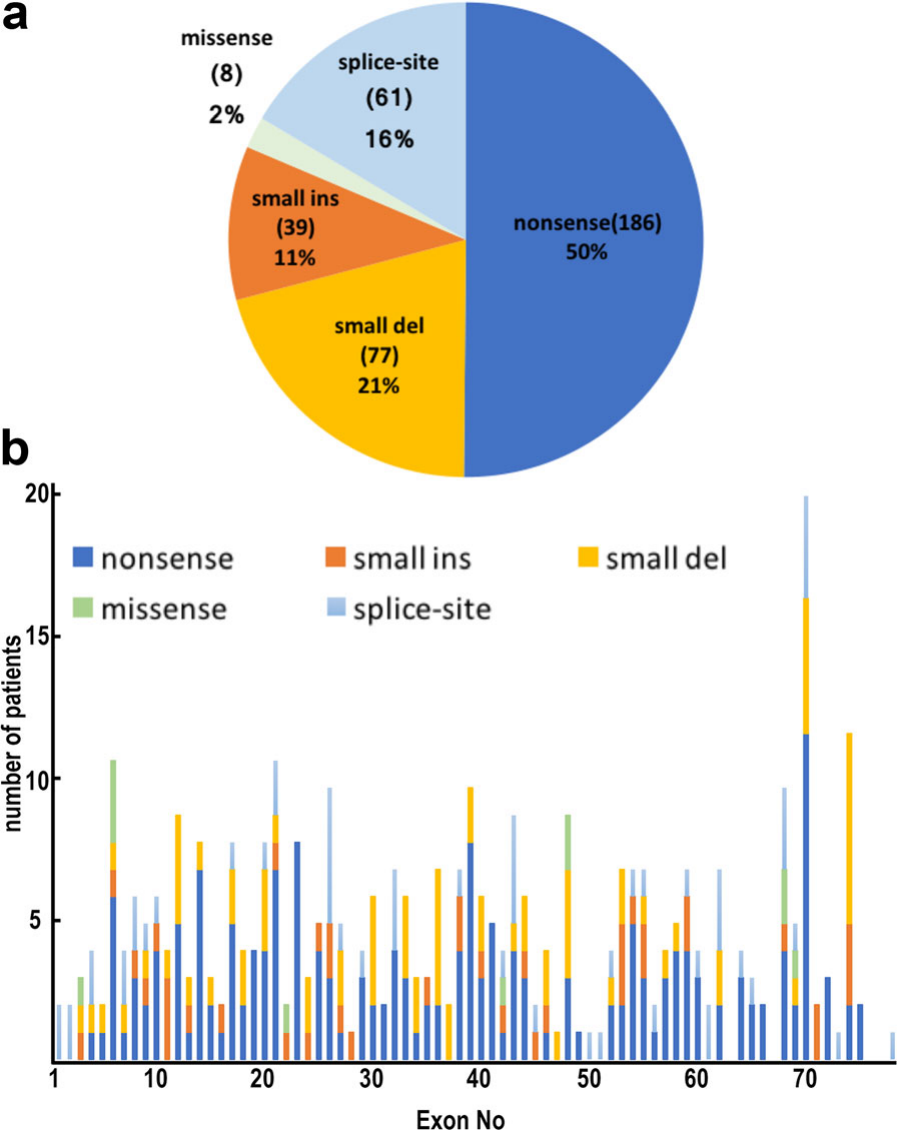
**a** Mutation spectrum of small mutations in the dystrophin gene in patients with dystrophinopathy. **b** Distribution of small mutations in *DMD*

The spectrum of identified mutations included nonsense mutations (186/371, 50%); small deletions (77/371, 21%); small insertions (39/371, 11%); splice-site mutations (61/371, 16%); and missense mutations (8/371, 2%) (Fig. 3a). The location of small mutations in patients with DMD and BMD patients is shown in Fig. 3b. Detailed results of the small mutations in the 371 patients, with 312 kinds of mutations, are shown in Additional file 1: Table S1. Among the 312 mutations, 149 were reported and 163 were unreported by HGMD (Human Gene Mutation Database) [18].

### Nonsense mutations

**Fig. 4:**
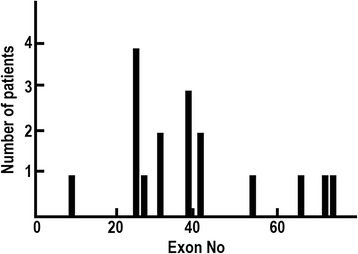
Distribution of the number of patients with nonsense mutations and faint and patchy dystrophin immunostaining

Nonsense mutations were the most common small mutations, accounting for 50% of the patients (186/371) (Fig. 3a). Although the mutations were almost uniformly distributed throughout the *DMD*, 6 patients shared the common mutation p.Arg3381* (c.10141C > T). Interestingly, 17 of 186 patients showed faint and patchy patterns of dystrophin immunostaining on skeletal muscles (Fig. 4, Additional file 1: Table S2). Clinical diagnosis of the 17 patients was DMD in 3 patients, IMD in 2, and BMD in 12, respectively.

### Missense variations

Only 8 patients (6 unrelated and 2 siblings) had 7 distinct missense variants (2% of the 371 patients with small mutations) (Table 1). Among them, 3 presented DMD phenotype while 3 BMD. Among the 7 patients who received muscle biopsy, the 2 patients showed total dystrophin deficiency, while 5 showed faint and patchy expression pattern on immunohistochemistry. None of the 7 missense variants was registered in any of HGVD, ExAC, and ESP6500. All variants were predicted to be probably or possibly damaging by PolyPhen2 (Table 1).

**Table 1:** Patients with DMD/BMD harboring missense mutations

Patient	Phetnotype	Dystrophin immunostaining	Exon	DNA change	Protein (predicted)	Frequency			PolyPhen2 prediction	
						HGVD	ESP6500	ExAC		
5	BMD	faint&patchy	3	c.152 T > G	p.Leu51Arg	no	no	no	0.999	probably
19	BMD	faint&patchy	6	c.434G > C	p.Arg145Pro	no	no	no	0.998	probably
22^*2^	BMD	not done	6	c.481A > C	p.Thr161Pro	no	no	no	0.628	possibly
23^*2^	BMD	faint&patchy	6	c.481A > C	p.Thr161Pro	no	no	no	0.628	possibly
112	DMD	negative	22	c.2949G > T	p.Gln9832His	no	no	no	0.991	probably
317	BMD	faint&patchy	68	c.9896A > G	p.His3299Arg	no	no	no	0.999	probably
320	DMD	faint&patchy	68	c.9937 T > C	p.Cys3313Arg	no	no	no	0.999	probably
325	DMD	negative	69	c.10011C > G	p.Cys3337Trp	no	no	no	1	probably

*2 represents brothers

## Discussion

This study retrospectively evaluated the results of gene analysis for 1497 patients with dystrophinopathies with clinical features and dystrophin immunostaining. Among the 1497 patients, 1092 (73%) were diagnosed by the MLPA method; including 901 patients with exon deletions (901/1497, 61%) and 188 patients with exon duplications (188/1497, 13%).

This is the report of the largest number of patients all previous dystrophinopathies reports from Asian countries [19–24]. Most of the previously reports from Asian countries [19–21] demonstrated lower deletion and higher duplication rates. This may be explained by the fact that much smaller numbers of patients were studied including female carriers. Nevertheless, Suh et al. [24], who reported the rates of deletions and duplications respectively, as 71.8%/ and 16.4% among 130 patients in Korea, which is similar not only to our results but also to the results reported from Western countries [25, 26]. In addition, exon deletion and duplication hotspots in our study (Fig. 2) are similar to those in the previous reports [20–22, 25, 26], suggesting that ethnicity is not a factor to cause any difference in the proportion of exon deletions and duplications in *DMD*.

Based on our results of exon deletions/duplications, we estimated the applicability of exon skipping therapies (Tables 2 and 3). Skipping of exon 51, which has been approved by FDA, and of exon 53 skipping, which is in clinical trials, cans theoretically be applied to the largest group of the patients with exon deletions, as suggested in previous reports [27].

**Table 2:** Overview of therapeutic exon 51 or 53 skipping for a series of DMD patients with exon deletion

skipped exon	Deleted exon	Number of patients
51	13–50	0
	29–50	0
	43–50	0
	45–50	23
	48–50	25
	49–50	29
	50	12
	52	18
	47–50	0
	52–63	0
		total 107
53	10–53	0
	43–52	0
	45–52	35
	47–52	0
	48–52	33
	49–52	19
	50–52	6
	52	18
		total 111

**Table 3:** Overview of the applicability of exon 51 or 53 skipping for DMD exon deletions

ekipping exon	Rate of applicable patients in all mutated patients	Rate of applicable patients in patients with exon deletion
51	7.1%	11.9%
53	7.4%	12.3%

Among 185 patients (13%) who harbor a nonsense mutation, interestingly, 17 patients showed faint and patchy dystrophin expression pattern on immunohistochemistry (Fig .4 and Additional file 1: Table S2). Nonsense mutations found in patients with BMD patients have reported to be present in exons 27, 29, 31, 37, 49, and 72 [28–30], all of which are in-frame exons, resulting in dystrophin positivity and milder phenotypes. Similarly 15 of the 17 patients with nonsense mutations and faint and patchy dystrophin staining in this study had mutations in in-frame exons. BMD phenotype in these patients may well be explained by the skipping of the correspondent exon, but cDNA analysis is necessary to be conclusive. From these results, there are 168 patients (12%) who are treated for nonsense codon read-through treatment.

Seven distinct missense variants were identified in 8 patients (Table 1). They may well be pathogenic since they are not reported in the HGVD, ESP6500, or ExAC and are predicted to be possibly or probably damaging in proteins by PolyPhen2. Especially, the 4 variants located in the N-terminus of dystrophin may be more likely to have pathogenic significance as previous reports showed the missense mutations in the actin binding domain of dystrophin, which is in the N-terminus region, induce thermodynamic instability of dystrophin molecules and protein aggregation [31, 32]. Needless to say, however, there still remains a possibility that patients may have unidentified causative mutations in other regions such as deep intronic regions or some missense mutations have an effect on splicing.

## Conclusion

Our report provides the largest *DMD* mutation dataset in Japan, which could be used as a reference for genetic diagnosis and will also help in further elucidating the nature of the disease.

## Additional files

Additional file 1: Table S1. Clinical and dystrophin immunostaining in the patients with a small mutation. **Table S2.** Patients with nonsense mutation and faint&patchy dystrophin staining. (PDF 286 kb)

## Abbreviations

BMD: Becker muscular dystrophy
DMD: Duchenne muscular dystrophy
ESP6500: Exome Sequencing Project
ExAC: Exome Aggregation Consortium
HGMD: Human gene mutation database
HGVD: Human Genetics Variation Browser
MLPA: Multiplex Ligation-dependent Probe Amplification
Remudy: Registry of Muscular Dystrophy
TREAT-NMD: Translational Research in Europe-Assessment and Treatment of Neuromuscular Diseases
