# Supplementary material for: Comprehensive analysis for genetic diagnosis of Dystrophinopathies in Japan
Source: Orphanet J Rare Dis. 2017 Aug 31;12:149. doi: 10.1186/s13023-017-0703-4 (PMC5580216; doi:10.1186/s13023-017-0703-4)
Supplement: Supplementary file 1 — Clinical and dystrophin immunostaining in the patients with a small mutation. Table S2. Patients with nonsense mutation and faint&patchy dystrophin staining. (PDF 286 kb) [file 13023_2017_703_MOESM1_ESM.pdf]

Table S1. Clinical and dystrophin immunostaining in the patients with a small mutation.

Patient	Phetnotype	Age (y)	Ambulatory	Age(y) at not ambulatory	Dystrophin immunostaining	Exon	DNA change	Protein (predicted)	Mutation function	Report
1	BMD	19	Yes		faint&patchy	int1	c.31+1G>T		splicing	reported
2	BMD	6	Yes		faint&patchy	int1	c.31+1G>T		splicing	reported
3	BMD	20	Yes		faint&patchy	int2	c.94-9dupT		splicing	unreported
4	BMD	17	Yes		faint&patchy	int2	c.94-1G>T		splicing	reported
5	BMD	64	No	56	faint&patchy	3	c.152T>G	p.Leu51Arg	missense	unreported
6	BMD	16	Yes		faint&patchy	3	c.157_158 delinsGA	p.Leu53Asp	small ins	unreported
7	BMD	37	No	24	faint&patchy	3	c.160_162 delCTC	p.Leu54del	small del	reported
8	DMD	3	Yes		not done	4	c.196A>T	p.Lys66*	nonsense	unreported
9	BMD	22	No	13	faint&patchy	4	c.238 delG	p.Ala80Hisfs*12	small del	unreported
10	BMD	10	Yes		faint&patchy	int4	c.264+1G>T		splicing	unreported
11	BMD	35	Yes		negative	int4	c.264+5G>A		splicing	unreported
12	BMD	23	Yes		faint&patchy	int4	c.265-463A>G		splicing	unreported
13	DMD	8	No		not done	5	c.312_313delTA	p.His104Glnfs*33	small del	unreported
14	BMD	22	Yes		negative	5	c.355C>T	p.Gln119*	nonsense	reported
15	DMD	9	Yes		not done	6	c.418delC	p.Leu140Serfs*2	small del	unreported
16	DMD	22	No	13	negative	6	c.428G>A	p.Trp143*	nonsense	unreported
17	DMD	5	Yes		negative	6	c.433C>T	p.Arg145*	nonsense	reported
18	DMD	18	No	8	negative	6	c.433C>T	p.Arg145*	nonsense	reported
19	BMD	32	No	16	faint&patchy	6	c.434G>C	p.Avg145Pro	missense	reported
20[*1]	DMD	9	Yes		negative	6	c.436C>T	p.Gln146*	nonsense	unreported
21[*1]	DMD	3	No		not done	6	c.436C>T	p.Gln146*	nonsense	unreported
22[*2]	BMD	33	No	17	not done	6	c.481A>C	p.Thr161Pro	missense	unreported
23[*2]	BMD	30	No	18	faint&patchy	6	c.481A>C	p.Thr161Pro	missense	unreported
24	DMD	32	No	8	not done	6	c.485_486dupGC	p.Trp163Alafs*7	small ins	unreported
25	DMD	9	Yes		negative	6	c.488G>A	p.Trp163*	nonsense	reported
26	DMD	12	Yes		negative	7	c.583C>T	p.Arg195*	nonsense	reported
27	DMD	13	No	9	negative	7	c.645delT	p.Glu216Lysfs*14	small del	unreported
28	DMD	11	Yes		negative	int7	c.649+1G>A		splicing	unreported
29	DMD	12	Yes		negative	int7	c.649+1 G>T		splicing	reported
30	DMD	13	Yes		negative	8	c.701C>G	p.Ser234*	nonsense	unreported
31	DMD	27	No	11	negative	8	c.724C>T	p.Gln242*	nonsense	reported
32	DMD	27	No	6	not done	8	c.724C>T	p.Gln242*	nonsense	reported
33	DMD	14	Yes		not done	8	c.783dupT	p.Lys262*	small ins	reported
34	BMD	17	Yes		negative	int8	c.831+1G>A		splicing	unreported
35	DMD	11	Yes		not done	int8	c.831+2T>C		splicing	unreported
36	DMD	8	Yes		negative	9	c.853G>T	p.Gly285*	nonsense	unreported
37	DMD	16	No	10	negative	9	c.857_858delAT	p.Tyr286*	small del	unreported
38	BMD	27	Yes		faint&patchy	9	c.883C>T	p.Arg295*	nonsense	reported
39	DMD	12	Yes		negative	9	c.899_903 CCTAC>TCTAG	p.Ala300_301Val*	nonsense	unreported
40	BMD	26	No	15	faint&patchy	int9	c.961-1G>A		splicing	reported
41	DMD	8	Yes		negative	10	c.966_967insT	p.Glu323*	small ins	unreported
42	DMD	27	No	11	negative	10	c.1061G>A	p.Trp354*	nonsense	reported
43	DMD	9	Yes		negative	10	c.1093C>T	p.Gln365*	nonsense	reported

44[*3]	DMD	15	Yes		negative	10	c.1117G>T	p.Glu373*	nonsense	reported
45[*3]	DMD	15	Yes		not done	10	c.1117G>T	p.Glu373*	nonsense	reported
46	DMD	4	Yes		negative	int10	c.1150-2A>T		splicing	unreported
47	DMD	7	Yes		negative	11	c.1201del C	p.Gln401fs*6	small del	unreported
48	DMD	18	No	9	negative	11	c.1205 dupT	p.Leu402Phefs*4	small ins	unreported
49	DMD	26	No	9	negative	11	c.1247_1248 insAGAAATGA	p.Thr418Metfs*10	small ins	unreported
50	DMD	16	Yes		not done	11	c.1323_1324delinsG	p.Gln442Lysfs*8	small ins	unreported
51	DMD	7	Yes		not done	12	c.1336_1337 del CA	p.His446*	small del	unreported
52	DMD	8	Yes		negative	12	c.1339A>T	p.Arg447*	nonsense	unreported
53	DMD	13	Yes		negative	12	c.1357C>T	p.Gln453*	nonsense	reported
54	DMD	6	Yes		negative	12	c.1365_1366delGA	p.Lys456Thrfs*6	small del	unreported
55	DMD	7	Yes		negative	12	c.1372A>T	p.Lys458*	nonsense	unreported
56	DMD	5	Yes		negative	12	c.1388G>A	p.Trp463*	nonsense	reported
57	DMD	5	Yes		negative	12	c.1394delC	p.Thy465Lysfs*22	small del	reported
58	DMD	22	No	9	negative	12	c.1429G>T	p.Gln477*	nonsense	reported
59	DMD	22	No	9	not done	12	c.1481_1482delAG	p.Lys494Serfs*24	small del	unreported
60	DMD	12	Yes		negative	13	c.1498delC	p.Leu500*	small del	unreported
61	DMD	20	No	12	negative	13	c.1504C>T	p.Gln502*	nonsense	unreported
62	IMD	12	Yes		negative	13	c.1603-15_1639dup52	p.Thr547Ilefs*16	small ins	unreported
63	DMD	9	Yes		negative	14	c.1615C>T	p.Arg539*	nonsense	reported
64	DMD	3	Yes		negative	14	c.1615C>T	p.Arg539*	nonsense	reported
65	DMD	15	Yes		not done	14	c.1627delA	p.Ile543Serfs*40	small del	reported
66	DMD	14	Yes		unknown	14	c.1652G>A	p.Trp551*	nonsense	unreported
67	DMD	20	No	9	negative	14	c.1663C>T	p.Gln555*	nonsense	reported
68	DMD	6	Yes		negative	14	c.1663C>T	p.Gln555*	nonsense	reported
69	DMD	5	Yes		negative	14	c.1663C>T	p.Gln555*	nonsense	reported
70	DMD	21	No	11	not done	14	c.1684C>T	p.Gln562*	nonsense	reported
71	DMD	6	Yes		not done	15	c.1707C>A	p.Cys569*	nonsense	reported
72	DMD	31	No	6	negative	15	c.1713delT	p.Phe571Leufs*12	small del	unreported
73	DMD	16	No	8	negative	15	c.1732A>T	p.Lys578*	nonsense	reported
74	DMD	19	No	10	negative	16	c.1952_1953delinsC	p.Trp651Serfs*4	small ins	unreported
75	DMD	16	No	8	negative	16	c.1966C>T	p.Gln656*	nonsense	reported
76	DMD	12	Yes		negative	17	c.2017C>T	p.Gln673*	nonsense	reported
77	DMD	41	No	9	not done	17	c.2032C>T	p.Gln678*	nonsense	reported
78	DMD	27	No	9	negative	17	c.2047G>T	p.Glu683*	nonsense	reported
79	DMD	21	No	9	negative	17	c.2061_2067del GGTGACC	p.Val688Glnfs*7	small del	unreported
80	DMD	12	Yes		negative	17	c.2069_2070delCA	p.Thr690Lysfs*29	small del	unreported
81	DMD	15	No	7	negative	17	c.2077C>T	p.Gln 693*	nonsense	reported
82	DMD	8	Yes		negative	17	c.2077C>T	p.Gln 693*	nonsense	reported
83	DMD	29	No	12	negative	int17	c.2169-1G>A		splicing	unreported
84	DMD	13	Yes		not done	18	c.2230_2231delAG	p.Pro745*	small del	reported
85[*4]	DMD	11	Yes		negative	18	c.2270C>G	p.Ser757*	nonsense	reported
86[*4]	DMD	8	Yes		not done	18	c.2270C>G	p.Ser 757*	nonsense	reported
87	DMD	7	Yes		negative	18	c.2281_2285delGAAAA	p.Glu761Serfs*10	small del	unreported
88	DMD	12	Yes		negative	19	c.2302C>T	p.Arg768*	nonsense	reported
89	DMD	12	Yes		negative	19	c.2302C>T	p.Arg768*	nonsense	reported

90	DMD	4	Yes		negative	19	c.2353C>T	p.Gln785*	nonsense	reported
91	DMD	43	No		not done	19	c.2365G>T	p.Glu789*	nonsense	reported
92	DMD	8	Yes		negative	20	c.2400delC	p.Ser800Argfs*9	small del	unreported
93	DMD	11	Yes		not done	20	c.2430_2443del14	p.Arg811Leufs*6	small del	reported
94	DMD	21	No	10	negative	20	c.2435G>A	p.Trp812*	nonsense	reported
95	DMD	20	No	11	unknown	20	c.2448C>A	p.Cys816*	nonsense	unreported
96	DMD	19	No	11	not done	20	c.2448C>A	p.Cys816*	nonsense	unreported
97	DMD	21	No	9	negative	20	c.2584G>T	p.Glu862*	nonsense	unreported
98	DMD	6	No		not done	20	c.2591delC	p.Thr864Lysfs*7	small del	reported
99	DMD	13	Yes		negative	int20	c.2622+1G>A		splicing	reported
100	DMD	31	No	10	unknown	21	c.2665C>T	p.Arg889*	nonsense	reported
101	DMD	7	Yes		faint&patchy	21	c.2673_2674 del AAinsG	p.Ile892Phefs*4	small ins	unreported
102	DMD	8	Yes		negative	21	c.2674delA	p.Ile892Phefs*4	small del	reported
103	BMD	19	Yes		negative	21	c.2704C>T	p.Gln902*	nonsense	reported
104	DMD	13	Yes		negative	21	c.2758C>T	p.Gln920*	nonsense	reported
105	DMD	20	No	12	unknown	21	c.2758C>T	p.Gln920*	nonsense	reported
106	DMD	17	No	9	negative	21	c.2758C>T	p.Gln920*	nonsense	reported
107	DMD	28	No	13	negative	21	c.2788A>T	p.Lys930*	nonsense	reported
108	DMD	17	No	8	negative	21	c.2797C>T	p.Gln933*	nonsense	reported
109	DMD	19	No	11	negative	int21	c.2804-1G>T		splicing	unreported
110	DMD	14	Yes		not done	int21	c.2804-2 A>G		splicing	reported
111	DMD	10	Yes		unknown	22	c.2808dupT	c.Asp937*	small ins	reported
112	DMD	7	Yes		negative	22	c.2949G>T	p.Gln9832His	missense	unreported
113	DMD	25	No	12	negative	23	c.2968C>T	p.Gln990*	nonsense	reported
114	DMD	16	Yes		negative	23	c.3013G>T	p.Glu1005*	nonsense	unreported
115	DMD	13	Yes		negative	23	c.3079 G>T	p.Gly1027*	nonsense	unreported
116	DMD	16	No	12	negative	23	c.3086G>A	p.Trp1029*	nonsense	reported
117	DMD	12	Yes		negative	23	c.3121C>T	p.Gln1041*	nonsense	reported
118	DMD	14	No	9	negative	23	c.3136C>T	p.Gln1046*	nonsense	reported
119	DMD	5	Yes		negative	23	c.3151C>T	p.Arg1051*	nonsense	reported
120	DMD	19	No	10	negative	23	c.3151C>T	p.Arg1051*	nonsense	reported
121	DMD	23	No	11	negative	24	c.3183delG	p.Lys1062Asnfs*10	small del	unreported
122	DMD	3	Yes		negative	24	c.3188_3200del	p.Trp1063Leufs*5	small del	unreported
123	DMD	14	Yes		negative	24	c.3257dupA	p.Gln1087Alafs*11	small ins	reported
124	IMD	19	Yes		faint&patchy	25	c.3295C>T	p.Gln1099*	nonsense	reported
125	BMD	8	Yes		faint&patchy	25	c.3304C>T	p.Gln1102*	nonsense	reported
126	BMD	31	Yes		faint&patchy	25	c.3337C>T	p.Gln1113*	nonsense	reported
127	DMD	24	No	9	negative	25	c.3408_3409delinsGT	p.Gln1137*	small ins	unreported
128	BMD	56	No	54	faint&patchy	25	c.3430C>T	p.Gln1144*	nonsense	reported
129	DMD	15	Yes		negative	26	c.3448G>T	p.Glu1150*	nonsense	unreported
130	DMD	15	Yes		negative	26	c.3500C>G	p.Ser1167*	nonsense	reported
131	DMD	22	No	9	negative	26	c.3530_3538 dup	p.Tyr1180*	small ins	unreported
132	DMD	19	No	9	not done	26	c.3562A>T	p.Lys1188*	nonsense	reported
133	DMD	15	Yes		negative	26	c.3565insA	p.Thr1189Asnfs*4	small ins	unreported
134	DMD	9	Yes		negative	int26	c.3603+1G>T		splicing	reported
135	BMD	32	Yes		faint&patchy	int26	c.3603+3A>T		splicing	reported
136	BMD	19	Yes		faint&patchy	int26	c.3603+3A>T		splicing	reported

137	BMD	23	Yes		faint&patchy	int26	c.3603+3A>T		splicing	reported
138	BMD	22	Yes		faint&patchy	int26	c.3603+3A>T		splicing	reported
139	DMD	9	Yes		faint&patchy	27	c.3613G>T	p.Glu1205*	nonsense	unreported
140	DMD	8	Yes		not done	27	c.3661_3662del	p.Val1221Lysfs*2	small del	unreported
141	DMD	9	Yes		negative	27	c.3734_3743 delACTACCA	p.Asn1246Serfs*7	small del	unreported
142	DMD	23	No	11	negative	27	c.3765dupT	p.Gly1256Trpfs*15	small ins	reported
143	DMD	29	No	10	unknown	int27	c.3786+1G>A		splicing	reported
144	BMD	44	Yes		faint&patchy	28	c.3909dupT	p.Glu1304*	small ins	reported
145	BMD	6	Yes		not done	29	c.3940C>T	p.Arg1314*	nonsense	reported
146	DMD	7	Yes		negative	29	c.4066G>T	p.Glu1356*	nonsense	unreported
147	DMD	4	Yes		not done	29	c.4066G>T	p.Glu1356*	nonsense	unreported
148	BMD	11	Yes		faint&patchy	int29	c.4071+2T>C		splicing	unreported
149	BMD	41	No	35	faint&patchy	30	c.4071_4079 delGGCTGTAAG	.Ala1358_Arg1360de	small del	unreported
150[*5]	DMD	19	No	11	negative	30	c.4087_4088 delAA	p.Lys1363Valfs*3	small del	unreported
151[*5]	DMD	16	No	11	not done	30	c.4087_4088 delAA	p.Lys1363Valfs*3	small del	unreported
152[*6]	DMD	20	No	10	negative	30	c.4126G>T	p.Glu1376*	nonsense	unreported
153[*6]	DMD	17	No	10	not done	30	c.4126G>T	p.Glu1376*	nonsense	unreported
154	DMD	33	No	10	negative	30	c.4160delC	p.Thr1387Asnfs*31	small del	unreported
155	BMD	14	Yes		faint&patchy	31	c.4294C>T	p.Gln1432*	nonsense	unreported
156	BMD	38	Yes		faint&patchy	31	c.4327C>T	p.Gln1443*	nonsense	unreported
157	DMD	10	Yes		not done	32	c.4375C>T	p.Arg1459*	nonsense	reported
158	DMD	17	No	8	negative	32	c.4384C>T	p.Gln1462*	nonsense	reported
159	DMD	8	Yes		negative	32	c.4495C>T	p.Gln1499*	nonsense	reported
160	DMD	14	Yes		negative	32	c.4501C>T	p.Gln1501*	nonsense	unreported
161	DMD	17	No	9	faint&patchy	int32	c.4518+1G>T		splicing	unreported
162	DMD	20	No	8	not done	int32	c.4518+2T>C		splicing	unreported
163	DMD	25	No	11	negative	int32	c.4518+5G>A		splicing	reported
164	DMD	14	Yes		negative	33	c.4535_4536delTG	p.Leu1512Glnfs*2	small del	unreported
165	DMD	18	No	12	negative	33	c.4537_4541delAGTGA	p.Lys1516*	small del	unreported
166	DMD	3	Yes		negative	33	c.4540G>T	p.Glu1514*	nonsense	unreported
167	DMD	25	No	11	negative	33	c.4545_4549delGAAGT	p.Lys1516*	small del	unreported
168[*7]	DMD	10	Yes		not done	33	c.4600C>T	p.Gln1534*	nonsense	reported
169[*7]	DMD	11	Yes		negative	33	c.4600C>T	p.Gln1534*	nonsense	reported
170[*8]	DMD	13	Yes		negative	34	c.4724delA	p.Lys1575Argfs*6	small del	unreported
171[*8]	DMD	11	No	7	not done	34	c.4724delA	p.Lys1575Argfs*6	small del	unreported
172	DMD	25	No	11	negative	34	c.4729C>T	p.Arg 1577*	nonsense	reported
173	DMD	14	No	6	unknown	35	c.4870 C>T	p.Gln1624*	nonsense	reported
174	DMD	35	No	10	not done	35	c.4996C>T	p.Arg1666*	nonsense	reported
175	DMD	28	No	7	unknown	35	c.5000_5001dupCA	p.Glu1668Glnfs*5	small ins	unreported
176	DMD	20	No	10	negative	36	c.5044G>T	p.Glu1682*	nonsense	reported
177	DMD	19	No	9	unknown	36	c.5052delT	p.Phe1684Leufs*37	small del	unreported
178	DMD	11	Yes		negative	36	c.5082G>A	p.Try1694*	nonsense	unreported
179	DMD	8	Yes		negative	36	c.5101delC	p.Leu1701Phefs*20	small del	unreported
180	DMD	15	No	10	negative	36	c.5131delC	p.Gln1711Serfs*10	small del	unreported
181	DMD	20	No	12	not done	36	c.5140delG	p.Glu1714Lysfs*7	small del	reported
182	IMD	49	No	13	not done	36	c.5140delG	p.Glu1714Lysfs*7	small del	reported

183	DMD	14	Yes		negative	37	c.5285delA	p.His1762Leufs*19	small del	unreported
184	DMD	22	No	11	negative	37	c.5285delA	p.His1762Leufs*19	small del	unreported
185	BMD	58	No	41	faint&patchy	38	c.5287C>T	p.Arg1763*	nonsense	reported
186	DMD	27	No	11	unknown	38	c.5350G>T	p.Glu1784*	nonsense	reported
187	DMD	16	Yes		negative	38	c.5364dupA	p.Asp1789Argfs*7	small ins	unreported
188	BMD	23	Yes		faint&patchy	38	c.5371C>T	p.Gln1791*	nonsense	reported
189	BMD	12	Yes		faint&patchy	38	c.5407C>T	p.Gln 1803*	nonsense	reported
190	DMD	5	Yes		negative	38	c.5413dupG	p.Val1805Glyfs*10	small ins	reported
191	DMD	11	Yes		negative	int38	c.5449-3 C>G		splicing	unreported
192	DMD	7	Yes		negative	39	c.5451_5454delTGAA	p.Asn1817Lysfs*7	small del	unreported
193	DMD	6	Yes		negative	39	c.5487_5488delAA	p.Gly1831Argfs*10	small del	reported
194	DMD	9	Yes		negative	39	c.5503C>T	p.Gln1835*	nonsense	unreported
195	DMD	6	Yes		negative	39	c.5530 C>T	p.Arg1844*	nonsense	reported
196	DMD	7	Yes		negative	39	c.5530C>T	p.Arg1844*	nonsense	reported
197[*9]	DMD	13	Yes		negative	39	c.5530C>T	p.Arg1844*	nonsense	reported
198[*9]	DMD	9	Yes		not done	39	c.5530C>T	p.Arg1844*	nonsense	reported
199	DMD	10	Yes		negative	39	c.5551C>T	p.Gln1851*	nonsense	reported
200	DMD	17	No	12	negative	39	c.5551C>T	p.Gln1851*	nonsense	reported
201	DMD	5	Yes		negative	39	c.5563C>T	p.Gln1855*	nonsense	reported
202	DMD	10	Yes		negative	40	c.5599C>T	p.Gln1867*	nonsense	unreported
203	DMD	14	Yes		negative	40	c.5602A>T	p.Arg1868*	nonsense	unreported
204	DMD	20	No	10	negative	40	c.5602_5605delAGAA	p.Arg1868Glnfs*5	small del	reported
205	DMD	13	Yes		negative	40	c.5602_5605delAGAA	p.Arg1868Glnfs*5	small del	reported
206	DMD	7	Yes		negative	40	c.5641C>T	p.Gln1881*	nonsense	reported
207	DMD	13	No	9	negative	40	c.5697dupA	p.Leu1900Ilefs*6	small ins	reported
208	DMD	7	Yes		negative	41	c.5758C>T	p.Gln1920*	nonsense	reported
209	DMD	9	Yes		negative	41	c.5770G>T	p.Glu1924*	nonsense	unreported
210	DMD	16	No	10	faint&patchy	41	c.5899C>T	p.Arg1967*	nonsense	reported
211	DMD	12	Yes		unknown	41	c.5899C>T	p.Arg1967*	nonsense	reported
212	IMD	12	Yes		faint&patchy	41	c.5899C>T	p.Arg1967*	nonsense	reported
213	DMD	17	Yes		negative	42	c.5985T>G	p.Tyr1995*	nonsense	reported
214	DMD	13	Yes		negative	42	c.6076_60778dupAA	p.Asp2027Argfs*13	small ins	unreported
215	BMD	16	Yes		faint&patchy	int42	c.6118-15A>G		splicing	unreported
216	DMD	29	No	10	not done	43	c.6136C>T	p.Gln2046*	nonsense	unreported
217	DMD	29	No	13	negative	43	c.6272delT	p.Met2091Serfs*23	small del	reported
218	DMD	16	Yes		negative	43	c.6283C>T	p.Arg2095*	nonsense	reported
219	DMD	26	No	9	negative	43	c.6283C>T	p.Arg2095*	nonsense	reported
220	DMD	7	Yes		negative	43	c.6283C>T	p.Arg2095*	nonsense	reported
221	DMD	24	No	11	negative	int43	c.6290+1G>T		splicing	unreported
222	BMD	24	No	18	faint&patchy	int43	c.6290+5G>C		splicing	unreported
223	DMD	23	No	10	negative	int43	c.6290+5G>C		splicing	unreported
224	DMD	17	Yes		not done	int43	c.6291-1G>A		splicing	reported
225	DMD	8	Yes		negative	44	c.6292C>T	p.Arg2098*	nonsense	reported
226[*10]	DMD	12	Yes		negative	44	c.6337delA	p.Ile2113*	small del	reported
227[*10]	DMD	10	Yes		not done	44	c.6337delA	p.Ile2113*	small del	reported
228	DMD	17	No	11	negative	44	c.6356G>A	p.Trp2119*	nonsense	unreported
229	DMD	16	Yes		not done	44	c.6430dupT	p.Tyr2144Leufs*3	small ins	reported

230	DMD	31	No	10	not done	44	c.6432T>A	p.Tyr3144*	nonsense	reported
231	DMD	15	Yes		negative	45	c.6611dupA	p.Arg2205Glufs*18	small ins	reported
232	IMD	22	No	12	faint&patchy	int45	c.6614+2T>C		splicing	reported
233	DMD	20	No	7	not done	int45	c.6615-2A>T		splicing	reported
234	DMD	13	No	8	negative	46	c.6643delG	p.Glu2215Asnfs*6	small del	unreported
235	DMD	25	No	11	negative	46	c.6659T>G	p.Leu2220*	nonsense	unreported
236	DMD	14	Yes		unknown	46	c.6751_6754delGAGC	p.Glu2252Lysfs*19	small del	unreported
237	DMD	12	Yes		negative	46	c.6754dupC	p.Gln2252Profs*21	small ins	unreported
238	DMD	19	No	10	not done	47	c.6804_6807delACAA	p.Lys2268Asnfs*2	small del	reported
239	DMD	5	Yes		negative	48	c.6913C>T	p.Gln2325*	nonsense	unreported
240	DMD	30	No	12	negative	48	c.6923_6933del11	p.Ala2308Glufs*6	small del	reported
241	DMD	20	No	11	not done	48	c.6949G>T	p.Glu2317*	nonsense	unreported
242	DMD	5	Yes		negative	48	c.6973C>T	p.Gln2325*	nonsense	reported
243	DMD	9	Yes		negative	48	c.6980delA	p.Lys2329Serfs*9	small del	unreported
244	DMD	25	No	7	negative	48	c.6986delA	p.Lys2329Serfs*9	small del	unreported
245	DMD	24	No	11	not done	48	c.7060delT	p.Tyr2354llefs*17	small del	unreported
246	DMD	10	Yes		negative	49	c.7126C>T	p.Gln 2376*	nonsense	unreported
247	IMD	37	No	13	faint&patchy	int50	c.7310-1G>A		splicing	reported
248	DMD	18	No	8	not done	int51	c.7543-5_7641del		splicing	unreported
249	DMD	24	No	12	not done	52	c.7565delA	p.Gln2522Argfs*15	small del	unreported
250	DMD	27	No	11	negative	52	c.7657C>T	p.Arg2553*	nonsense	reported
251	DMD	17	Yes		not done	52	c.7657C>T	p.Arg2553*	nonsense	reported
252	DMD	8	Yes		negative	int52	c.7661-11T>C		splicing	unreported
253	DMD	16	Yes		negative	53	c.7665_7666 ins GA	p.Arg2556Glufs*21	small ins	unreported
254	DMD	15	Yes		negative	53	c.7686_7702del17insA	p.Asp2562Glufs*9	small ins	unreported
255	DMD	6	Yes		negative	53	c.7693C>T	p.Gln2565*	nonsense	reported
256	DMD	25	No	8	negative	53	c.7714delA	p.Arg2572Glyfs*4	small del	unreported
257	DMD	24	No	10	unknown	53	c.7750C>T	p.Gln2584X*	nonsense	unreported
258	DMD	9	Yes		not done	53	c.7787delT	p.Leu2596*	small del	unreported
259	DMD	14	Yes		negative	53	c.7833dupT	p.Thr2612Tyrfs*31	small ins	unreported
260	DMD	23	No	10	negative	54	c.7873C>T	p.Gln2625*	nonsense	unreported
261	DMD	16	No	9	negative	54	c.7891dupC	p.Arg2631Profs*12	small ins	unreported
262[*11]	DMD	10	Yes		negative	54	c.7894C>T	p.Gln2632*	nonsense	unreported
263[*11]	DMD	13	Yes		negative	54	c.7894C>T	p.Gln2632*	nonsense	unreported
264	BMD	13	Yes		faint&patchy	54	c.8009G>A	p.Trp 2670*	nonsense	reported
265	DMD	35	No	9	unknown	54	c.8010G>A	p.Trp2670*	nonsense	unreported
266	DMD	14	Yes		negative	int54	c.8027+11C>T		splicing	unreported
267[*12]	DMD	15	No	8	not done	55	c.8053G>T	p.Glu2685*	nonsense	unreported
268[*12]	DMD	18	No	10	negative	55	c.8053G>T	p.Glu2685*	nonsense	unreported
269	DMD	6	Yes		not done	55	c.8159delG	p.Arg2720Leufs*6	small del	unreported
270	DMD	13	Yes		negative	55	c.8171_8172insT	p.Leu2725Profs*20	small ins	unreported
271	DMD	15	Yes		negative	55	c.8209_8210 dup CA	p.Gln2737Hisfs*28	small ins	unreported
272	DMD	6	Yes		negative	55	c.8214G>A	p.Trp2738*	nonsense	reported
273	DMD	28	No	10	negative	int55	c.8217+5G>A		splicing	unreported
274	DMD	17	No	12	negative	int55	c.8218-2A>G		splicing	reported
275	DMD	10	Yes		negative	56	c.8299G>T	p.Glu2767*	nonsense	unreported
276	DMD	19	No	11	negative	int56	c.8391-1G>A		splicing	unreported

277	DMD	18	No	11	negative	57	c.8416C>T	p.Gln2806*	nonsense	reported
278	DMD	16	Yes		negative	57	c.8438delC	p.Ser2813Phefs*11	small del	unreported
279	DMD	14	Yes		negative	57	c.8459G>A	p.Trp2820*	nonsense	unreported
280	DMD	13	Yes		negative	57	c.8479G>T	p.Glu2827*	nonsense	unreported
281	DMD	13	Yes		negative	58	c.8560G>T	p.Glu2854*	nonsense	unreported
282	DMD	4	Yes		not done	58	c.8606C>T	p.Arg2876*	nonsense	unreported
283	DMD	9	Yes		not done	58	c.8608C>T	p.Arg2870*	nonsense	reported
284	DMD	8	No		not done	58	c.8608C>T	p.Arg2870*	nonsense	reported
285	DMD	7	Yes		negative	58	c.8652_8653delCT	p.Tyr2885Profs*9	small del	unreported
286	DMD	7	Yes		negative	59	c.8692C>T	p.Gln2898*	nonsense	reported
287[*13]	BMD	31	No	20	not done	59	c.8713C>T	p.Arg2905*	nonsense	reported
288[*13]	DMD	34	No	12	not done	59	c.8713C>T	p.Arg2905*	nonsense	reported
289	DMD	17	No	8	negative	59	c.8713C>T	p.Arg2905*	nonsense	reported
290	DMD	14	No	8	negative	59	c.8821_8822 insAGGCCACTTCAAG	p.Asp2944Glyfs*6	small ins	unreported
291	DMD	19	No	11	negative	59	c.8833_8854dup	p.Gln2952Argfs*10	small ins	unreported
292	DMD	12	Yes		negative	int59	c.8669-1 G>C		splicing	reported
293	DMD	14	Yes		negative	60	c.8944C>T	p.Arg2982*	nonsense	reported
294	DMD	11	Yes		negative	60	c.8944C>T	p.Arg2982*	nonsense	reported
295	DMD	12	Yes		negative	60	c.9036T>A	p.Tyr3012*	nonsense	unreported
296	DMD	37	No	12	negative	int60	c.9085 -1 G>A		splicing	unreported
297	DMD	35	No	11	negative	int61	c.9163+1G>A		splicing	reported
298	DMD	14	Yes		negative	int61	c.9164-2A>G		splicing	unreported
299	DMD	14	Yes		negative	62	c.9173delA	p.Gln3058Argfs*31	small del	unreported
300	DMD	7	Yes		negative	62	c.9183G>A	p.Trp3061*	nonsense	reported
301	DMD	4	Yes		negative	62	c.9204_9207delCAAA	p.Asn3068Lysfs*20	small del	reported
302	DMD	13	No	8	not done	62	c.9216C>G	p.Tyr3072*	nonsense	unreported
303	DMD	12	Yes		negative	int62	c.9225-10T>A		splicing	unreported
304	DMD	13	Yes		not done	int62	c.9225-287C>A		splicing	unreported
305	DMD	26	No	11	not done	int62	c.9225-2A>C		splicing	unreported
306	DMD	6	No		not done	64	c.9337C>T	p.Arg3113*	nonsense	reported
307	DMD	2	No		not done	64	c.9337C>T	p.Arg3113*	nonsense	reported
308	BMD	1	No		not done	64	c.9337C>T	p.Arg3113*	nonsense	reported
309	DMD	13	Yes		negative	int64	c.9361+1G>A		splicing	reported
310	DMD	9	Yes		negative	65	c.9522C>A	p.Cys3174*	nonsense	reported
311	DMD	5	Yes		negative	65	c.9524G>A	p.Trp3180*	nonsense	reported
312	DMD	17	Yes		negative	int65	c.9563+1G>A		splicing	reported
313[*14]	DMD	9	Yes		faint&patchy	66	c.9568C>T	p.Arg3190*	nonsense	reported
314[*14]	DMD	4	No		not done	66	c.9568C>T	p.Arg3190*	nonsense	reported
315	DMD	32	No	19	negative	68	c.9816dupT	p.Lys3273*	small ins	reported
316	DMD	16	Yes		not done	68	c.9851G>A	p.Trp3284*	nonsense	reported
317[*15]	BMD	31	Yes		faint&patchy	68	c.9896A>G	p.His3299Arg	missense	unreported
318[*15]	DMD	27	No	8	not done	68	c.9928C>T	p.Gln3310*	nonsense	reported
319[*15]	DMD	30	No	9	not done	68	c.9928C>T	p.Gln3310*	nonsense	reported
320	DMD	19	No	10	faint&patchy	68	c.9937T>C	p.Cys3313Arg	missense	unreported
321	DMD	16	No	6	negative	68	c.9948C>A	p.Cys3316*	nonsense	unreported
322	DMD	9	Yes		faint&patchy	int68	c.9975-2A>G		splicing	reported

323	DMD	18	No	11	unknown	int68	c.9974+1G>C		splicing	unreported
324	DMD	12	Yes		negative	int68	c.9975-2A>G		splicing	unreported
325	DMD	7	Yes		negative	69	c.10011C>G	p.Cys3337Trp	missense	reported
326	DMD	25	No	9	negative	69	c.10033C>T	p.Arg3345*	nonsense	reported
327	DMD	13	Yes		negative	69	c.10033C>T	p.Arg3345*	nonsense	reported
328	DMD	16	No	10	negative	69	c.10086delG	p.Thr3363Leufs*14	small del	unreported
329	DMD	6	Yes		negative	int69	c.10087-2A>C		splicing	unreported
330	DMD	7	Yes		not done	70	c.10096 G>T	p.Gly3366*	nonsense	unreported
331	DMD	35	No	9	not done	70	c.10096_10098delGGA	p.Gly3366del	small del	unreported
332	DMD	7	Yes		negative	70	c.10108C>T	p.Arg3370*	nonsense	reported
333	DMD	14	No	6	negative	70	c.10108C>T	p.Arg3370*	nonsense	reported
334	DMD	12	Yes		negative	70	c.10137delA	p.Lys3379Asnfs*23	small del	unreported
335	DMD	8	Yes		negative	70	c.10141C>T	p.Arg3381*	nonsense	reported
336	DMD	20	No	10	negative	70	c.10141C>T	p.Arg3381*	nonsense	reported
337	DMD	9	Yes		negative	70	c.10141C>T	p.Arg3381*	nonsense	reported
338	DMD	8	Yes		not done	70	c.10141C>T	p.Arg 3381*	nonsense	reported
339	DMD	9	Yes		not done	70	c.10141C>T	p.Arg3381*	nonsense	reported
340	DMD	42	No	10	not done	70	c.10141C>T	p.Arg3381*	nonsense	reported
341[*16]	DMD	14	Yes		not done	70	c.10171C>T	p.Arg3391*	nonsense	reported
342[*16]	DMD	20	No	8	unknown	70	c.10171C>T	p.Arg3391*	nonsense	reported
343	DMD	13	Yes		negative	70	c.10171C>T	p.Arg3391*	nonsense	reported
344	DMD	21	No	8	not done	70	c.10174delA	p.Met3392Trpfs*10	small del	unreported
345	DMD	9	Yes		not done	70	c.10174delA	p.Met3392Trpfs*10	small del	unreported
346	DMD	8	Yes		negative	70	c.10216_10223 delATGGAAAC	p.Met3406Serfs*24	small del	unreported
347	DMD	15	Yes		negative	int70	c.10223+1G>A		splicing	reported
348	DMD	19	No	8	negative	int70	c.10223+3G>C		splicing	reported
349	DMD	12	Yes		negative	int70	c.10223+6T>A		splicing	unreported
350	DMD	21	No	11	not done	int70	c.10224-1insT		splicing	unreported
351	BMD	57	Yes		faint&patchy	71	c.10231dupA	p.Thr3411Asnfs*22	small ins	reported
352	BMD	11	Yes		faint&patchy	71	c.10231dupA	p.Thr3411Asnfs*22	small ins	reported
353	BMD	43	Yes		faint&patchy	72	c.10279C>T	p.Gln3427*	nonsense	reported
354[*17]	IMD	43	No	15	not done	72	c.10320T>A	p.Tyr3440*	nonsense	unreported
355[*17]	DMD	42	No	18	negative	72	c.10320T>A	p.Tyr3440*	nonsense	unreported
356	BMD	35	Yes		faint&patchy	int73	c.10395-1 G>C		splicing	unreported
357	BMD	7	Yes		faint&patchy	74	c.10402G>T	p.Glu3468*	nonsense	unreported
358	DMD	11	Yes		faint&patchy	74	c.10406dupA	p.His3469Glnfs*22	small ins	reported
359	DMD	9	Yes		not done	74	c.10453dupC	p.Leu3485Profs*6	small ins	reported
360	DMD	6	Yes		negative	74	c.10453dupC	p.Leu3485Profs*6	small ins	reported
361	BMD	37	No	17	negative	74	c.10453_10454delCT	p.Leu3485Glufs*5	small del	unreported
362	BMD	37	No	25	unknown	74	c.10453_10454delCT	p.Leu3485Glufs*5	small del	unreported
363	DMD	6	Yes		negative	74	c.10454delT	p.Leu3485Argfs*11	small del	reported
364	DMD	27	No	11	not done	74	c.10454delT	p.Leu3485Argfs*11	small del	reported
365	DMD	5	Yes		negative	74	c.10495G>T	p.Glu3499*	nonsense	unreported
366[*18]	BMD	18	Yes		faint&patchy	74	c.10498_10499delAG	p.Ser3500*	small del	reported
367[*18]	BMD	9	Yes		not done	74	c.10498_10499delAG	p.Ser3500*	small del	reported
368	DMD	14	Yes		negative	74	c.10500_10501delTG	p.Ser3500Argfs*14	small del	unreported

369	DMD	23	No	13	negative	75	c.10651C>T	p.Gln3551*	nonsense	reported
370	BMD	22	Yes		not done	75	c.10792G>T	p.Glu 3598*	nonsense	unreported
371	BMD	4	Yes		not done	int78	c.10554-18C>G		splicing	reported

The patients with *same number are brothers.

Table S2. Patients with nonsense mutation and faint&patchy dystrophin staining

no	phenotype	dystrophin immunostain	exon	DNA change	protein (predicted)
38	BMD	faint&patchy	9	c.883C>T	p.Arg295*
124	IMD	faint&patchy	25	c.3295C>T	p.Gln1099*
125	BMD	faint&patchy	25	c.3304C>T	p.Gln1102*
126	BMD	faint&patchy	25	c.3337C>T	p.Gln1113*
128	BMD	faint&patchy	25	c.3430C>T	p.Gln1144*
139	DMD	faint&patchy	27	c.3613G>T	p.Glu1205*
155	BMD	faint&patchy	31	c.4294C>T	p.Gln1432*
156	BMD	faint&patchy	31	c.4327C>T	p.Gln1443*
185	BMD	faint&patchy	38	c.5287C>T	p.Arg1763*
188	BMD	faint&patchy	38	c.5371C>T	p.Gln1791*
189	BMD	faint&patchy	38	c.5407C>T	p.Gln 1803*
210	DMD	faint&patchy	41	c.5899C>T	p.Arg1967*
212	IMD	faint&patchy	41	c.5899C>T	p.Arg1967*
264	BMD	faint&patchy	54	c.8009G>A	p.Trp 2670*
313	DMD	faint&patchy	66	c.9568C>T	p.Arg3190*
353	BMD	faint&patchy	72	c.10279C>T	p.Gln3427*
357	BMD	faint&patchy	74	c.10402G>T	p.Glu3468*
